# Eco-evolutionary agriculture: Host-pathogen dynamics in crop rotations

**DOI:** 10.1371/journal.pcbi.1007546

**Published:** 2020-01-16

**Authors:** Maria Bargués-Ribera, Chaitanya S. Gokhale

**Affiliations:** Research Group for Theoretical Models of Eco-evolutionary Dynamics, Department of Evolutionary Theory, Max Planck Institute for Evolutionary Biology, Plön, Germany; University of California Irvine, UNITED STATES

## Abstract

Since its origins, thousands of years ago, agriculture has been challenged by the presence of evolving plant pathogens. Temporal rotations of host and non-host crops have helped farmers to control epidemics among other utilities, but further efforts for strategy assessment are needed. Here, we present a methodology for developing crop rotation strategies optimal for control of pathogens informed by numerical simulations of eco-evolutionary dynamics in one field. This approach can integrate agronomic criteria used in crop rotations—soil quality and cash yield—and the analysis of pathogen evolution in systems where hosts are artificially selected. Our analysis shows which rotation patterns perform better in maximising crop yield when an unspecified infection occurs, with yield being dependent on both soil quality and the strength of the epidemic. Importantly, the use of non-host crops, which both improve soil quality and control the epidemic results in similar rational rotation strategies for diverse agronomic and infection conditions. We test the repeatability of the best rotation patterns over multiple decades, an essential end-user goal. Our results provide sustainable strategies for optimal resource investment for increased food production and lead to further insights into the minimisation of pesticide use in a society demanding ever more efficient agriculture.

## Author summary

The invention of agriculture is a major evolutionary transition in the social evolution of the human race. Transforming the lifestyle from nomadic to sedentary, agriculture provided humankind with the stability necessary to make rapid advancements. However, agriculture, as we know it, is now in danger. While agriculture is a grand artificial selection experiment, it is in a constant battle with the brute force of natural selection, generating highly infectious plant pathogens. Traditional techniques such as slash-burn techniques are not sustainable for feeding the ever-increasing population. Crop rotation, on the other hand, has been developed over thousands of years as a sustainable method. We provide a computational model of how crop rotations can be used to tackle pathogen infection and what properties of rotation patterns make them sustainable in the long run. We hope that this study, together with other sustainable methods such as minimal pesticide use and biocontrol, can make agriculture more efficient.

## Introduction

Around ten thousand years ago, changes in climate conditions led to the emergence of agricultural practices in human hunter-gatherer communities around the globe [1]. This process of domestication—or artificial selection—was refined along centuries through trial and error, combined with experience, increasing the quantity and quality of the product. In the case of plant agriculture, the presence of pests has been a substantial threat to effective production [2]. The first farmers already tried to overcome the pest problem by employing field rotations, i.e., shifting cultivation techniques [3–5], among other methods. As the human population continues to multiply, current agriculture practices need to address a two-fold problem of the dearth of enough food supply and plant pathogens. Techniques such as slash-and-burn, pesticides and fertilisers are used for increasing yield as well as dealing with pests but do not contribute to agricultural sustainability [6]. Thus, current research needs to focus on developing cropping techniques which increase yield and mitigate the environmental impact [7]. Nowadays, data-based computational tools are used to design agricultural strategies. Among others, the computational tools involve decision support models for choosing optimal cropping plans—which cultivar to grow and where—and crop rotation decisions [8, 9]. The models guide allocating crops depending on their characteristics—botanical family, market demand, or soil demands –, examining the spatial distribution and temporal successions. However, these models need to integrate other farming concerns, one of which being the control of plant pathogens [10].

In evolutionary biology, models on host-pathogen coevolution have contributed to understanding the relationship between some plant pathogens and their hosts in natural ecosystems, for example, regarding the specificity of the interaction [11, 12]. In domesticated crops, pathogen evolution is driven by natural selection in tandem with artificial selection: hosts do not coevolve with the pathogen but, are instead, bred according to human interests. Recently, authors have highlighted the use of plant-pathogen evolutionary theory in formulating disease management strategies and avoiding the increase of infectivity in pathogens [13–17]. Regarding evolutionary dynamics, theoretical approaches have studied trade-offs in plant-pathogen evolution linked to a periodic absence of host crops [18], a situation which resembles the phenomena in crop rotations. When rotating, a change in the crop type acts as a perturbation leading to frequent selective sweep-like dynamics. Tracking the frequency and speed of such sweeps would be useful in detecting periods of lower fitness and reduced population size; in which the pathogen could be pushed to extinction [19]. Then, adjusting the models used in natural plant-pathogen coevolution to the study of crop rotations can be a useful approach for tackling agricultural problems.

In this manuscript, we aim to design a cultivating strategy optimal for pathogen damage control, integrating agronomic criteria—soil quality and yield—used on crop rotations and pathogen evolution depending on the cultivated host. Our model for crop rotations focuses on patterns which maximise yield and appoint soil quality as a variable of interest. When infection occurs, ecological dynamics play out in the short term. Host-pathogen dynamics predicts crop loss depending on host susceptibility, as well as changes in pathogen load. We study pathogen evolution by including a transition of the pathogen into strains which can infect the host more efficiently. From all possible crop rotation patterns, only a few are good at maximising crop yield. Such patterns are shared among some tested pathosystems which have different characteristics. In general, an abundance of cover crop seasons is required as they play a double-role in both improving soil quality and breaking the epidemic. Knowledge of the initial soil status is shown to be vital in determining the actual best rotation. The field can only afford a 1:1 cash-cover crop ratio if nutrients are in excess from the beginning. The rotation patterns which maximise cash yield are assessed according to their performance over ten years, but also their ability to be used in a second and third decade. For sustainability over more extended periods, maintenance of soil quality and the minimisation of pathogen evolution into more infectious strains become central.

Overall, our computational model provides a generic framework which can be, at the interest of the researcher or farmer, adapted to particular plant-pathogen case studies. It provides guidelines, and it helps to understand the utility of crop rotations in pest management from an eco-evolutionary perspective.

## Models and methods

Plants have a variety of responses to pest infestations such as susceptibility, tolerance and resistance. In agriculture, farmers have used this variability for thousands of years to control the spread of pathogens. This simple yet powerful concept is formalised below, using a theoretical model which analyses the effect of rotation patterns in infection dynamics.

### Between-season model description: Optimising rotations from soil quality

To establish a basic model of rotation patterns, we focus on a sequential combination of cash crops and cover crops. Cash crops are those which provide a product to be commercialised—e.g. maize—whereas cover crops improve the soil quality of the field but provide no direct, substantial cash yield—e.g. clover. Since better soil quality provides more cash yield, including both crop types can result in improved farming: the basic model aims to study which temporal patterns of cash and cover crops maximise the farmer’s benefit. We have been inspired by a previous report assessing the optimal length of clover period, compared to maize, in a 9-year field experiment [20]. Here we work with pattern sequences of length *L* = 10 seasons to acquaint long-term patterns with increased optimisation [21].

We explore the space of possible cash-cover combinations of *L* = 10 exhaustively and attribute each of the 2^*L*^ rotation sequences a yield value *Y* which consists in the yield accumulated at the end of the ten seasons.

**Fig 1:**
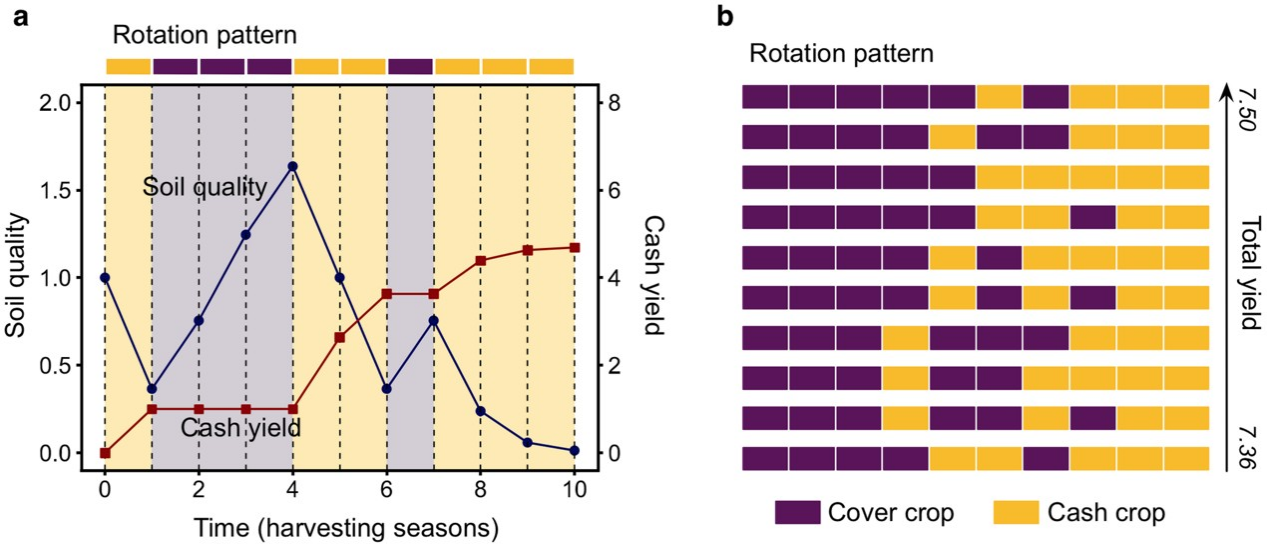
Soil quality and cash yield variations in a rotation sequence and selection of 10 sequences that maximise cash yield. a) Each time step corresponds to a harvesting season. Dots indicate discrete values for soil quality (blue circles) and cash yield (red squares). Season crop type is indicated by yellow (lighter) for cash crops and purple (darker) for cover crops. b) Ten optimal rotation patterns according to total cash yield *Y*. Each row is a rotation sequence, ordered from maximum to minimum yield (top to bottom) among the selection.

Each element in a rotation sequence corresponds to a harvesting season, modelled as a discrete time-step. During each time-step *t*, there is a change in soil quality *q*(*t*) and cash yield *y*(*t*), which varies depending on the crop type *i* = {1, 2} (cash crops: *i* = 1, cover crops: *i* = 2.) (Fig 1). The change in soil quality and cash yield, per time-step, are crucial in obtaining the final yield, taking the following form:

- Soil quality (*q*(*t*)): Soil quality decreases following a logistic decay curve for cash crops *c*_1_ and increases with logistic growth for cover crops *c*_2_. The parameter *β*_*i*_ regulates the intensity and direction of the soil quality change given crop type *i* at time *t*. We set *β*_1_ = −1.5 for soil quality decrease by *c*_1_, and *β*_2_ = 1 for soil quality increase by *c*_2_, considering that it is more difficult to improve soil quality than to decrease it (see S1 Appendix for further examples). We assume that the soil quality cannot increase indefinitely, reaching a saturation value (carrying capacity) of *K*. We choose *K* = 2, for which approximately *n*_2_ = 4 harvesting seasons are needed to reach it with *β*_2_ = 1, similar to the observations of a field report [20]. In the logistic function, when values are very close to the upper and lower limits, the change is minimal both in growth and decay; hence we set thresholds of maximum *q*(*t*) = 1.99 and minimum *q*(*t*) = 0.01 from which soil quality does not vary, so changes become more perceptible. Also, the initial soil quality is set to *q*(0) = 1, assuming the median value,
  $$\begin{array}{c} q\left(t+1\right)=max[0.01,min(1.99,\frac{Kq\left(t\right)e^{\beta_{i}}}{K+q\left(t\right)(e^{\beta_{i}}-1)})]. \end{array}$$ (1)
- Cumulative cash yield (*y*(*t*)): Cash yield increases in proportion to the soil quality at the beginning of the season *q*(*t*), regulated by the crop yield contribution *γ*_*i*_. For cash crops, we set *γ*_1_ = 1, making cash yield increase in proportion to the soil quality, in a 1:1 ratio. For cover crops, there is no cash yield increase, *γ*_2_ = 0 (see S1 Appendix for further crop characterisation information). The cash yield accumulates along the rotation sequence time-steps,
  $$\begin{array}{c} y\left(t+1\right)=y\left(t\right)+\gamma_{i}q\left(t\right). \end{array}$$ (2)
- Total yield (*Y*): This is simply the value of *y*(*t*) evaluated at *t* = *L*. We define it separately since it is used as the main criteria to compare sequences and assess their optimality.

The time series of each possible rotation pattern in a population of sequences of length *L* = 10 are computed according to the above-defined functions and analysed.

To understand the model predictions, we focus first on the top ten sequences whose patterns maximise cash yield and, hence, have a higher *Y* (Fig 1b). These sequences share investment in—mostly—three consecutive cash crops during the last seasons, and they have the same number of cash and cover seasons. This information could be interesting for farmers to maximise their economic output, but it has two drawbacks: it does not take into account how the rotations perform under the real threat of pathogens and, not surprisingly, the soil quality by the end of the rotation is almost completely degraded. We assess each of these concerns in turn.

### Within-season dynamics: Adding eco-evolutionary dynamics

In natural settings, the process of coevolution between the host plant and its pathogen can lead to the cyclic evolution of host resistance and parasite virulence, maintaining genetic diversity [15, 22] In agriculture, humans are the selecting agent: they decide which host grows in the next generation. While being economically significant, the selected crop can be particularly vulnerable to pathogens, which it has not been exposed to before. Moreover, there are only a few major agricultural cash crops; resulting in less genetic diversity in host crops and more disease susceptibility for the cultivars not selected for resistance [23]. In this section, we show how the introduction of a pathogen affects the assessment of a rotation sequences optimality. We start with a simple infection scenario in which a pathogen *p* can infect cash crops *c*_1_, but not the cover crops *c*_2_, using the second as break crops [24]. As an example, the fungi *Fusarium graminearum* [25] is one such pathogen which infects cash crops like maize or wheat but does not infect cover crops such as clover.

#### Ecological dynamics

To include host-pathogen ecological dynamics, we adapt the Lotka-Volterra competitive equations, based on [26]. Within a season, time is continuous, and dynamics described by a system of two ordinary differential equations,

$$\dot{c_{i}}=-c_{i}\sigma_{i}\sum_{j}^{}p_{j}$$ (3)

$$\dot{p_{j}}=p_{j}(\sum_{i}^{}\sigma_{i}c_{i}-d_{j}).$$ (4)

While the equations can allow for multiple hosts and pathogens, we start with two types of crop host (*i* = {1, 2}) and a single pathogen (*j* = 1). Here *c*_*i*_ is the population density of crop *i* and *p*_*j*_ is the pathogen density. The pathogen infectivity is set by *σ*_*i*_ (*σ*_1_ = 0.04 for the susceptible cash crop, *σ*_2_ = 0 for the cover resistant crop), and *d*_*j*_ is the death rate of the pathogen (*d*_*j*_ = 0.5). Due to the artificial setting of agriculture, we consider that without external perturbations and except for the pathogen-induced mortality, there is no birth nor death in the host population during the season. The host density declines when the pathogen is present according to its infectivity *σ*_*i*_. This decline could be more complicated if we would include host tolerance with a pathogen density-dependent function regulating *σ*_*i*_. In this study, we use a straightforward approach.

**Fig 2:**
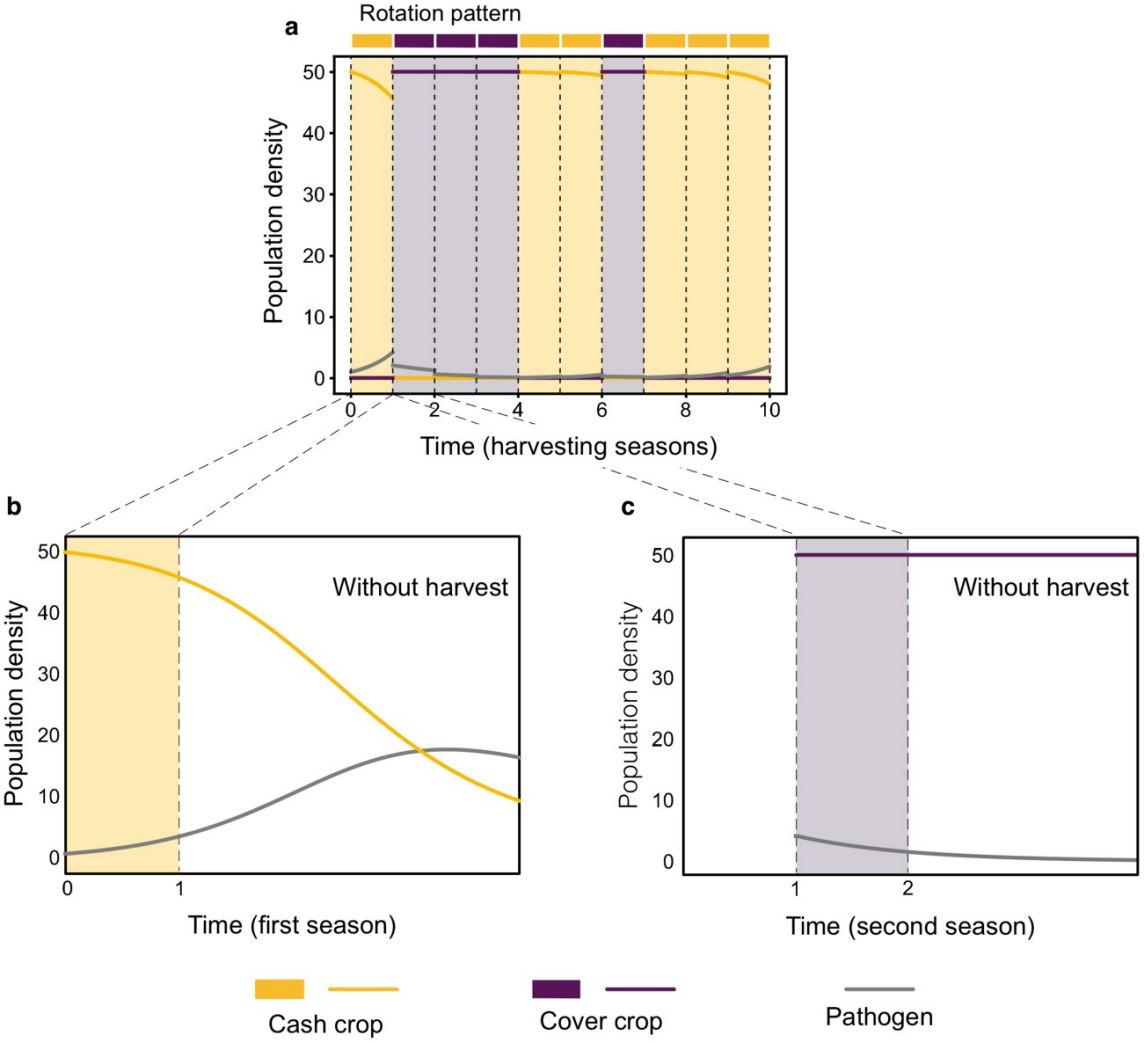
Host-pathogen ecological dynamics, within and between seasons. a) Dynamics between seasons. After each harvest, initial host density (*h*(0) = 50) is reinitialised and pathogen density is readjusted according to the pathogen retainment (*ϵ* = 0.5). b) Dynamics within a season, when there is a susceptible cash crop (*i* = 1). Host density decreases due to the presence of the pathogen, while the pathogen load increases as long as there are enough crops to infect. c) Dynamics within a season, when there is a non-host cover crop (*i* = 2). The cover crop maintains its output while remaining unaffected by the pathogen. The pathogen dies since it cannot grow on the cover crop. Both b) and c) show how the dynamics would continue without the harvest.

Change from one season to the next is a discrete-time step. At the end of each season, the crop is harvested, converted to yield, and new crops planted as per the rotation schedule. While harvesting, the pathogen population is disturbed. Some pathogens survive in soil or residues of the infected crops (pathogen retainment *ϵ*). We set *ϵ* = 0.5, considering that half of the pathogen population stays in the field. Overall, the model evolves following continuous-time within seasons, and discrete jumps between seasons, being an example of a hybrid dynamical system [27] as used for seasonal plant epidemiology [28, 29] (Fig 2).

#### Eco-evolutionary dynamics

Substantial evolutionary changes can happen on ecological time-scales [30–32]. Consequently, we need to include evolution in the host-pathogen interaction. In our case, we study the dynamics when there is the evolution of pathogen virulence, and incorporate them in the already developed ecological dynamics. In literature, virulence refers to the pathogen capacity to establish an infection or the consequences for the host to be infected [33]. We focus on the propensity of a pathogen to damage the host, through the regulation of *σ*_*i*_. Within a season, the pathogen reproduces, generates variation, and some of these variants may carry mutations that provide more virulence. We do not include any costs for the additional infectivity. To incorporate evolution in the ecological dynamics, we modify the previous Eq (3) allowing the pathogen to mutate into strains which can exploit the cash host more efficiently, Eq (5). The evolved pathogen cannot evolve to infect the cover crop since cash and cover are assumed to be phylogenetically distant and the cover is then a non-host species [34],

$$\begin{array}{l} \begin{array}{ccc} \dot{c_{i}} & = & -\sum_{j}W_{ji}\sigma_{i}c_{i}p_{j} \end{array}\\ \begin{array}{ccc} \dot{p_{j}} & = & \sum_{k}Q_{jk}p_{k}\sum_{i}W_{ki}\sigma_{i}c_{i}-p_{j}d_{j}. \end{array} \end{array}$$ (5)

The new equations have two critical elements: the transition matrix *Q*_*kj*_ and the fitness matrix *W*_*ji*_. The transition matrix *Q*_*kj*_ corresponds to the rates in which the pathogen can mutate between five possible strains. The strains are separated from each other by unit genetic distance. Thus to reach *p*_5_ the original strain requires four mutational steps. Mutation can happen between strains which are one mutational step away with a transition rate *μ* = 0.1,

$$Q_{jk}=\begin{array}{l} p_{1}\\ p_{2}\\ p_{3}\\ p_{4}\\ p_{5} \end{array}(\begin{array}{ccccc} p_{1} & p_{2} & p_{3} & p_{4} & p_{5}\\ 1-\mu & \mu & 0 & 0 & 0\\ \mu & 1-2\mu & \mu & 0 & 0\\ 0 & \mu & 1-2\mu & \mu & 0\\ 0 & 0 & \mu & 1-2\mu & \mu \\ 0 & 0 & 0 & \mu & 1-\mu \end{array}).$$ (6)

**Fig 3:**
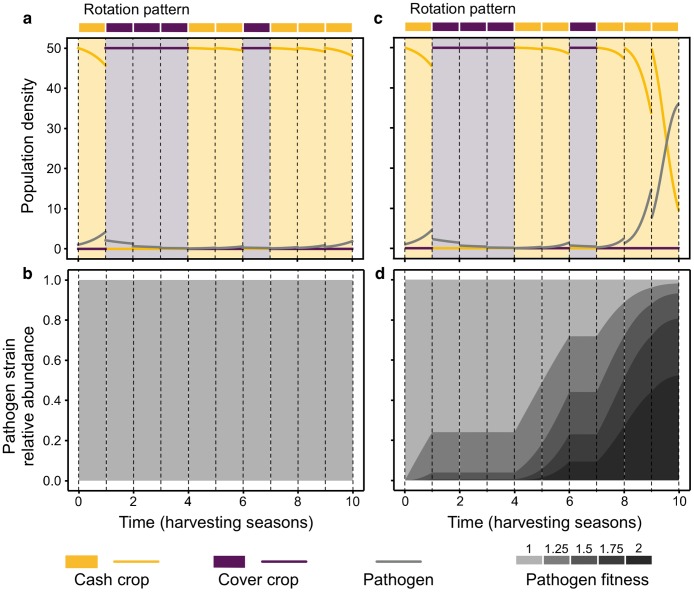
Only ecological vs. eco-evolutionary dynamics of host-pathogen interaction. a) Ecological dynamics, without pathogen evolution. Dynamics between seasons are represented, with infection starting at *t* = 0 (as in Fig 2a). b) The pathogen population is homogeneous, due to the absence of mutation. c) Eco-evolutionary dynamics with pathogen virulence evolution. Dynamics between seasons are represented, with infection starting at *t* = 0. Due to pathogen evolution (with *μ* = 0.1), the impact of the infection in the last cash seasons provokes higher host density loss, compared to a). d) Time evolution of pathogen shows that the relative abundance of fitter strains—in darker colours—increases along seasons. In both b) and d), relative abundances of the pathogen strains are plotted.

For the fitness matrix *W*_*ji*_, we set the fitness of the original pathogen *p*_1_ to *w*_11_ = 1 when infecting *c*_1_; and to *w*_12_ = 0 when infecting *c*_2_. Each mutant increases the fitness proportional to the distance with respect to *p*_1_, so *w*_*j*1_ = *w*_11_ + 0.25(*j* − 1), with *w*_51_ = 2 being the maximum fitness in our example system with five pathogen genotypes. Infecting *c*_2_ does not provide any fitness benefit, with *w*_*j*2_ = 0. The fitness matrix, when multiplied by the parameter *σ*_*i*_, shows the increase in virulence in each mutated strain of the pathogen. In general, eco-evolutionary dynamics results in elevated crop loss as compared to the solely ecological dynamics (Fig 3).

### Coupling eco-evolutionary dynamics with yield loss

When modelling the eco-evolutionary dynamics, pathogen growth decreases crop density. Therefore, those seasons which suffer infection do not have a cash yield outcome equivalent to their healthy counterparts. Consequently, the total yield *Y* by which we choose the best sequences changes its value the more the crop is infected. To estimate the loss of crop yield, we modify the cash yield to consider the host density at the time of harvest: the effective crop ratio, or *δ*(*t*). It indicates the proportion of the host population that is uninfected at the end of the season (dividing the crop density at the end of the season by the initial density),

$$\begin{array}{c} \delta \left(t+1\right)=\frac{c_{i}\left(t+1\right)}{c_{i,t}}\\ y\left(t+1\right)=y\left(t\right)+\delta \left(t+1\right)\gamma_{i}q\left(t\right). \end{array}$$ (7)

Included in the equation of cash yield, Eq (7), *δ*(*t*) modifies the outcome of the season, so only uninfected crops contribute to the yield. Because even infected crops take nutrients from the soil, we do not include *δ*(*t*) in the soil quality equation.

All the parameters used in this modelling framework are collated in Table 1 and described with justification in the Supporting Information (see S1 Appendix). The code corresponding to the model implementation is available online.

**Table 1:** List of fixed parameters used in the model.

Parameter	Description	Value	Reference
*β*_*i*_	Soil contribution of host *i*	*β*_1_ = −1.5, *β*_2_ = 1	
*γ*_*i*_	Cash contribution of host *i*	*γ*_1_ = 1, *γ*_2_ = 0	
*K*	Carrying capacity of the soil	*K* = 2	
*μ*	Transition rate	*μ* = 0.1	
*H*	Initial host density	*H* = 50	
*σ*_*i*_	Infectivity of pathogen for host *i*	*σ*_1_ = 0.04,*σ*_2_ = 0	[12, 26]
*d*_*j*_	Death rate of the pathogen *p*_*j*_	*d*_*j*_ = 0.5	[12, 26]

## Results

### Optimal rotation patterns under infection: The protective effect of cover crops

**Fig 4:**
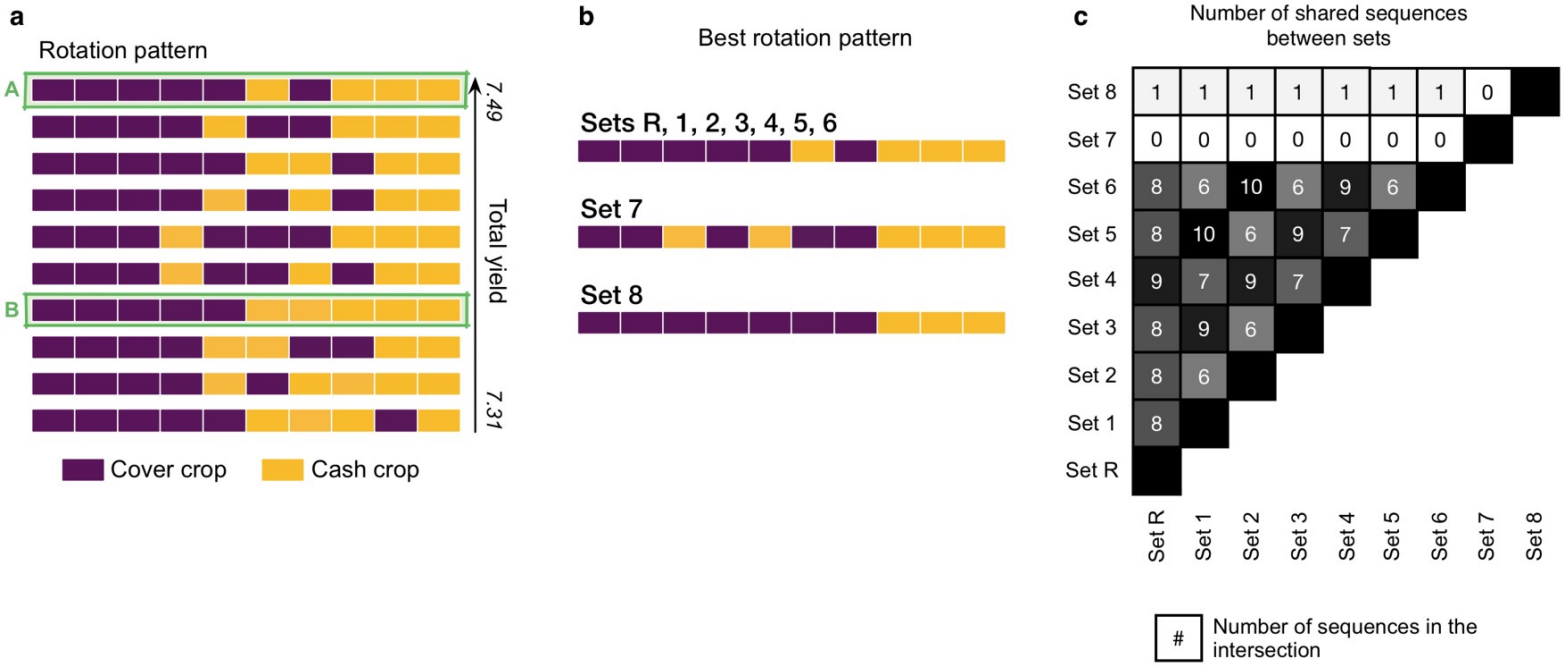
Best patterns under infection in different conditions. a) Selection of ten best patterns from 1024 possible sequences when cash yield loss due to infection is computed using the reference values. Each row is a rotation sequence. b) Best rotation sequence in the set of 10 optimal patterns for each of the conditions. The set index corresponds to conditions as indicated in Table 2. c) Intersection array for the sets of optimal sequences under different conditions. Each cell shows the number of sequences found in the intersection between the sets indicated in the vertical and horizontal labels. Highlighted sequences in (a): We allow for the 1024 possible sequences to repeat twice or thrice i.e. two or three generations. Rotation A is the sequence that maximises yield over multiple generations while Rotation B maximises yield only in the first generation but not later on.

Using the effective crop ratio *δ*(*t*) we compute the values of total yield *Y* for each sequence. We model the scenario in which the pathogen infects at the beginning of the first season, at *t* = 0, and include pathogen evolution. As previously, we focus on the ten rotation patterns which yield the best (Fig 4a). Interestingly, results show that 8 out of 10 rotation patterns which have a greater *Y* in the presence of the pathogen coincide with the set of rotations that maximise yield in pathogen-free conditions.

Within the set of 10 optimal sequences, the yield range is 7.50 ≤ *Y* ≤ 7.36 without infection and reduces to 7.49 ≤ *Y* ≤ 7.31 with infection. In both sets, the best rotation pattern is the one starting with five seasons of cover crop, alternating after that and ending with three cash seasons. The reason behind the coincidence of patterns between the two sets is the double effect that the cover crops provide: on the one hand, they increase soil quality which in turn increases yield; on the other hand, they break the epidemic diminishing crop loss and minimising yield loss.

### Sensitivity of optimal patterns to different pathogen and soil conditions

Neither all epidemics have the same intensity, nor do all fields respond the same under the same farmer’s practices. Here we explore the conditions under which our set of rotations can maximise yield and compare it with the sets of rotations which have a better outcome in other scenarios. By a set, we refer to the selection of 10 optimal sequences among the 1024 possible rotation patters. We also compare the maximum value of cash yield that we can get for each condition (Table 2).

**Table 2:** Yield and crop ratio for different pathogen and soil conditions. Sets refer to the selection of 10 sequences which best maximise yield in each condition. Values in bold indicate the change of conditions in the set with respect to the reference set.

Sequence set	Initial pathogen *p*_1_(0)	Pathogen retainment *ϵ*	Initial soil quality *q*(0)	Initial pathogen fitness *w*_11_(0)	Maximum yield *Y*_*max*_	Cash:cover ratio
Reference (R)	1	0.5	1	1	7.49	2:3,1:1
Set 1	1	**0.8**	1	1	6.93	2:3
Set 2	1	**0.2**	1	1	7.50	1:1,2:3
Set 3	**10**	0.5	1	1	7.38	2:3
Set 4	**0.1**	0.5	1	1	7.50	2:3,1:1
Set 5	1	0.5	1	**1.5**	6.91	2:3
Set 6	1	0.5	1	**0.5**	7.50	1:1,2:3
Set 7	1	0.5	**1.9**	1	9.29	1:1
Set 8	1	0.5	**0.1**	1	5.30	3:7, 2:3

#### Pathogen retainment

Crop rotations are used to control the disease, but not all pathogens are equally vulnerable to the effects of break crops, here cover crops. The spores of airborne pathogens, such as fungi, can disperse over long distances and are difficult to control with crop rotations because the infection often spreads from the neighbouring fields. Conversely, crop rotations can be beneficial for soil-borne pathogens which cannot reproduce on a non-host plant [4, 35]. The ability of pathogens to survive in the soil or in crop debris, which can also be modified by tillage practices, is represented in our model by the pathogen retainment (*ϵ*). In the previous simulations, *ϵ* = 0.5, and here we explore what happens if its value increases to *ϵ* = 0.8 and decreases to *ϵ* = 0.2.

When we increase the retainment (set 1), the maximum yield decreases to *Y* = 6.93, and the optimal sequences have a ratio of two cash crops for every three cover crops in all cases. The number of cover seasons increases because there is a need for a more extended period of non-host crops to compensate that more pathogen stays in the soil. When we decrease the retainment (set 2), the maximum yield is approximately maintained, being *Y* = 7.50., and also the ratio of cash and cover crops.

#### Initial pathogen inoculum

For the pathogens, the characteristics of the initial inoculum can determine the severity of the epidemic [36]. Here we explore it in two ways: the quantity of pathogen in the initial inoculum (*p*_1_(0)) and the initial virulence of the pathogen, controlled by the values in the fitness matrix (*W*_*ij*_) at time *t* = 0. The default initial pathogen in our model is *p*_1_(0) = 1; here, we observe how a ten-fold increase *p*_1_(0) = 10 and decrease *p*_1_(0) = 0.1 affect the optimal rotation patterns and yield. For the pathogen fitness, we conserve the ability of the pathogen to mutate into five fitter strains, but we set values of *w*_11_ = 1.5 and *w*_11_ = 0.5 as initial fitness, in comparison to the reference *w*_11_ = 1 (with *w*_*j*2_ = 0, as before, for the cover crops *c*_2_).

Starting with an initial pathogen of *p*_1_(0) = 10 (set 3) decreases the maximum yield to *Y* = 7.38 and decreases the ratio of cash to cover crops to 2:3 in all the sequences. The decrease is not drastic since starting with five consecutive cover crops decreases the pathogen load. This feature is present also in the reference set, to increase soil quality, showing the double effect of the cover crops. The decrease of inoculum (set 4) maintains the yield to *Y* = 7.50 and keeps the reference crop ratio. The increase of pathogen fitness (set 5) reduces the yield to *Y* = 6.91 and decreases the cash to cover ratio to 2:3. The results of decreasing pathogen fitness (set 6) are similar to the decrease of initial inoculum, being the yield *Y* = 7.50 and the ratio maintained to 1:1 or 2:3.

#### Initial soil quality

When farmers aim to maximise cash yield, disregarding soil quality can lead to a sterile field which needs more cover crops than *a priori* expected. Since the rotation plan may start in a field with poor quality, we check the effect of initial soil quality on the patterns. The values chosen are *q*(0) = 1.9, close to the carrying capacity *K* = 2, and *q*(0) = 0.1.

High initial soil quality (set 7) leads to the highest maximum yield increase, being *Y* = 9.29 and the ratio of crops 1:1. This yield increase is because we can get the highest yield in the first seasons, and we can maintain soil quality by the alternation of crops (Fig 4b). The number of cash crops cannot increase more because this would promote the infection, decreasing the yield. Low initial soil quality (set 8) has the most substantial reduction of maximum yield, decreasing to *Y* = 5.30. Dedicating more seasons in improving soil quality at the beginning, the ratio of cash to cover crops decays to 3:7 or 2:3 (Fig 4b).

#### Intersection of optimal sets

Results show that the set of 10 best sequences shown in the previous section—and chosen as reference set—intersects with the optimal sets obtained in all conditions except for increased initial soil quality, despite changes in the maximum yield. We check for the number of common rotation sequences via a pairwise comparison of the sets for each of the exposed conditions (Fig 4c). The cases for which the sets intersect the most with the reference set relate to the initial pathogen: increase and decrease of pathogen retainment *ϵ* (8/10), increase (8/10) or decrease (9/10) of initial pathogen *p*_1_(0) and changes of initial pathogen fitness *w*_11_(0) (8/10). When pathogen retainment and pathogen fitness is high, there is full intersection due to a common need for more non-host crops that break the epidemic (Set 1 and Set 5, 10/10); and also vice-versa (Set 2 and Set 6, 10/10). Other conditions also have high intersection values between them (from 6/10 to 9/10) due to similar needs for both increasing soil quality and controlling the infection. Variations in soil quality lead to the most different optimal patterns, with low (1/10) or no intersection with the rest of the sets.

### Longer-term rotations: Soil quality and virulence control for the next generation of crops

Ten seasons, or a decade in yearly crops, can be regarded as long term planning, but farmers cultivate fields for even longer. To investigate if our rotation patterns are sustainable over decades, we study the variation in the yield and the pathogen load in consecutively repeated patterns.

For the analysis, we focus on the repetition of the population of sequences of length *L* = 10 i.e. all of the 1024 possible patterns and then repeating them twice or thrice. We term these repetitions as generations. We do not explore, however, the complete combinatorial space presented by the inclusion of second and third generation (i.e. 2^20^ or 2^30^ combinations), which is beyond the scope and focus of this manuscript. Of the 1024 patterns we limit our attention to the sets of 10 sequences that best maximise the yield in the infection scenario of reference (*p*_1_(0) = 1, *ϵ* = 0.5, *w*_11_(0) = 1) and median initial soil quality (*q*(0) = 1).

The results show that the rotations that best maximise yield after the second and third generation coincide with the optimal rotations for the first generation (intersection of 8/10 for both sets). To further investigate their sustainability, we analyse the changes of the agronomic variables—soil quality and cash yield—and the host-pathogen eco-evolutionary dynamics. We focus on two rotation patterns: the common optimal rotation for all generations Fig 4 (rotation A) and a rotation from the 10-optimal set of the first generation which is excluded in the set for the second and third generations Fig 4 (rotation B). Rotation A starts with five cover crops, alternates for two seasons and finishes with three cash crops; rotation B has five cover crops followed by five cash crops.

The analysis (Table 3) shows that rotation A maintains the initial soil quality after the 10th season (*q*(10) = 1), while rotation B depletes it (*q*(10) = 0.15). In the previous section, we have shown that initial soil quality is key in determining the optimal rotation. Because of this feature, rotation A is able to maintain its optimal performance in the following generations, but B would need more investment in soil quality to aim for the same cash yield. Importantly, pathogen evolution during the first generation is also determinant in the yield outcome in the future. For rotation A, the increased frequency of virulent pathogen strains (*f*(*p*_5_) = 0.28) provokes more yield loss during the infection time. Consequently, the cash yield within the second (*Y* = 7.38) and third (*Y* = 6.32) generation is lower than within the first instance (*Y* = 7.49), even if soil quality is maintained. This effect is more drastic for rotation B, which initiates the second generation with high frequency of virulent strains (*f*(*p*_5_) = 0.46) and shows severe infection when a cash crop is cultivated. The frequency of *p*_5_ is chosen to be an indicator for virulence. If the strain *p*_5_ exists then the existence of all other strains is guaranteed.

**Table 3:** Performance of rotation A and B along three generations. A and B are the highlighted sequences in Fig 4. Rotation A is the sequence that maximises yield over multiple generations. Rotation B maximises yield in the first generation but not in the subsequent. For each rotation and generation there are shown values for total yield, final soil quality and final frequency of the most virulent strain (*p*_5_).

	1st generation			2nd generation			3rd generation		
	Y	*q*(10)	*f*(*p*_5_)(10)	Y	*q*(20)	*f*(*p*_5_)(30)	Y	*q*(30)	*f*(*p*_5_)(30)
A	7.49	1	0.28	7.38	1	0.65	6.32	1	0.67
B	7.36	0.15	0.46	3.72	0.01	0.66	1.23	0.01	0.67

**Fig 5:**
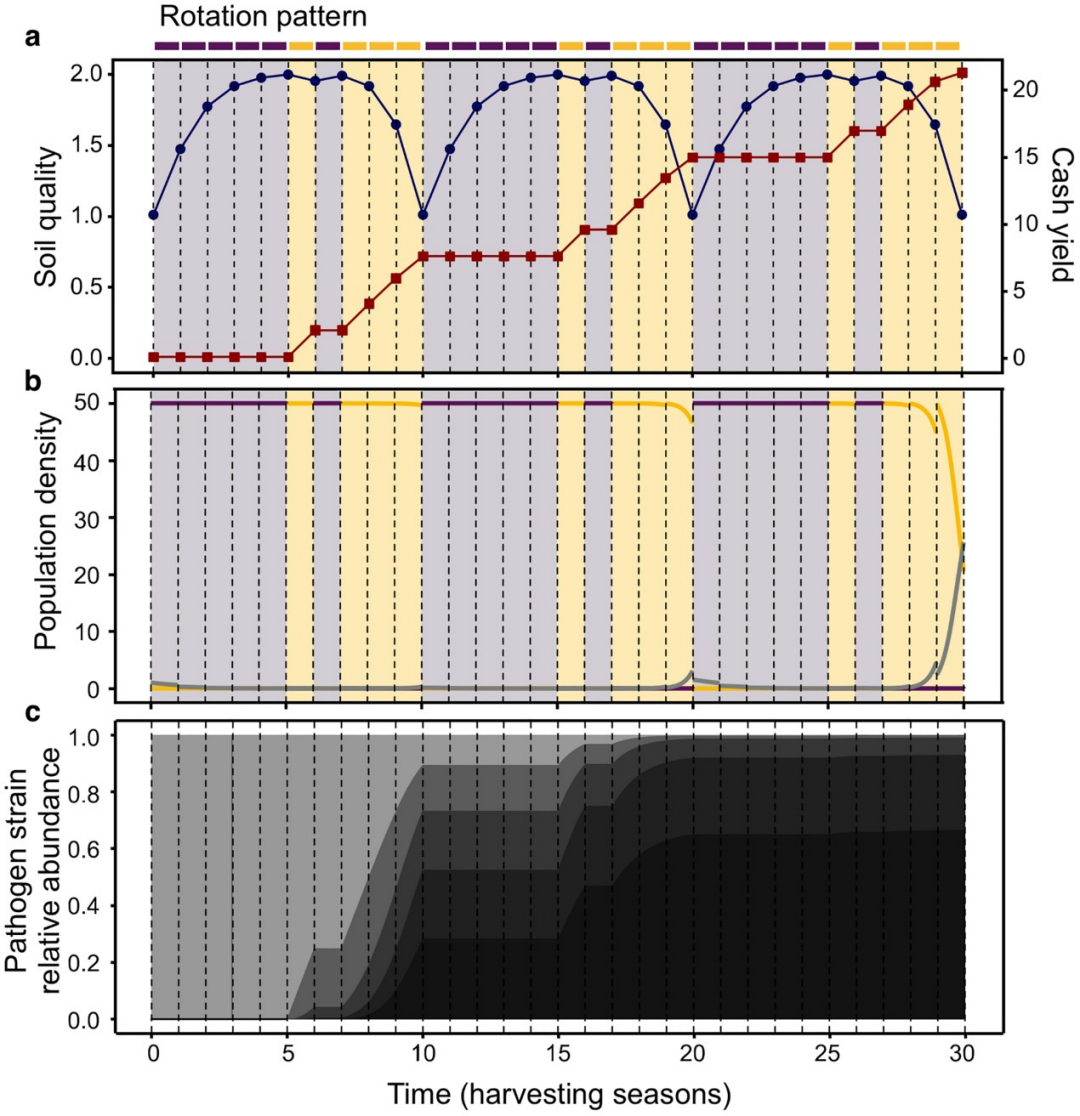
Eco-evolutionary dynamics of rotation A when repeated thrice (30 seasons). A) Soil quality (blue circles) and cash yield (red squares) variations, in discrete time-steps which correspond to the harvesting seasons. B) Eco-evolutionary dynamics of crop (yellow = cash, purple = cover) and pathogen (grey) within and between seasons. C) Relative abundances of pathogen strains during the rotation.

Remarkably, the pathogen strain with more fitness does not outcompete the rest of strains (Fig 5). Since pathogens can mutate in both forward and reverse directions with the same rate (Eq (5)), the system reaches a mutation-selection balance in which the rate of generating strains with less fitness equals the rate at which the fitter strains are generated. The faster growth of the fitter strains is reflected in their higher eventual frequency in equilibrium.

These results show the properties of the rotation patterns that maintain soil quality and slow down pathogen evolution in the long term—requirements for sustainable farming.

## Discussion

Translational evolutionary biology is a growing field where fundamental concepts from evolutionary biology can be used in an applied setting to make effective changes in society [37, 38]. Just as with the search for novel antibiotics, the search for novel agricultural strategies can benefit immensely from evolutionary biology. Notably, applying evolutionary principles can help pest management in agroecosystems. Our work complements previous attempts on coupling plant genetics with resistance deployment strategies [17, 39], but with a new focus on plant-pathogen dynamics and pathogen evolution in the context of crop rotation sequences.

We present a model for assessing how different patterns of cash and cover crop rotations influence long-term yield outcome and soil quality. Reported computational tools [8, 9, 40] rely on historical data to predict which is the best decision. Typically farmers take a number of different factors (e.g. social, economical, biological and practical) into account when deciding upon an agricultural strategy. Our model indeed simplifies this complex decision-making process by choosing to focus on a smaller set of parameters such as soil quality, cash yield, infection dynamics and pathogen virulence evolution. In such a controlled setting, we are thus able to provide a *de novo* assessment integrating features of the crop field, epidemiology and pathogen evolution. Our results highlight that only a few patterns, from all possible crop rotation sequences, can maximise the yield. The resulting patterns suggest, broadly, investing in soil quality during the first seasons and once close to the carrying capacity of the soil, alternating the cultivation of cash and cover crops. By the end of the rotation, investment in cash crops maximises the yield of the decade.

During the harvesting seasons, pathogens may invade the field and damage the crops, diminishing the expected yield. Using plant-pathogen dynamics, we have tracked the ecology and evolution of the infection in discrete and continuous time, predicting the possible magnitude of infection for each rotation sequence. By modelling pathogen ecology and evolution, we apply evolutionary biology concepts to agricultural strategies, as done in previous theoretical models [17, 41], but we couple the dynamics with yield loss along with our rotations, to re-assess which rotations perform the best under infection. The resulting patterns coincide with the ones obtained under the no infection scenarios. The alternation of non-host cover crops with susceptible cash crops allows for efficient epidemic control and also to increase soil quality (and thus the yield), even in the absence of infection.

The rotations that maximise the yield depend on the conditions of the field and the epidemic. However, across several parameter values in our model, we observe consistency among the best patterns. These parameters are relevant for representing different plant pathosystems, as they characterise the initial pathogen, its retainment between seasons and its virulence for the host plant. The congruence in the rotation sequences could be significant for the farmers wishing to mitigate epidemics, when uncertain about the soil status or especially the presence of quiescent pathogens in the field. Based on a field history report, the initial conditions can be tuned to a specific rotation plan, thus adapting the model to desired crop characteristics for particular plant-pathogen case studies.

The patterns constrained by a limited time horizon always dedicate the final seasons to cash crops, depleting soil quality. However, in the long run, maintaining the levels of soil quality is necessary to have similar conditions after each rotation pattern, bringing the possibility of reapplying the sequence. The analysis of repetition of patterns for a second and third decade shows that investing more in cover crops is critical for long-term yield output. Acknowledging foresight, we are promoting the sustainability criterion [42] in our system. The conditions presented for the first decade become similar to the ones that future decades will find so that the strategies can be maintained—perhaps with some variations due to external factors, e.g. climate.

From a sustainability point of view, besides soil quality, the capacity of the pathogen to evolve is also critical. In agriculture, most of the crop pathogens evolve rapidly, due to high planting density and genetic uniformity of the host, which increase the effective population size leading to more frequent random variation in the population [43]. Several well-known commercial varieties of crops suffer from such problems, such as the Cavendish bananas affected by *Fusarium* wilt, also known as Panama disease [44]. The strategies presented in this study do not eradicate the infection, but some rotations can delay the growth of more virulent strains. In the results, the sequences which yield the most in the second and third decade also have a slower increase of frequency of the virulent strains. The knowledge from our model could be coupled to current research that works on cultivar mixtures and crop mosaic patterning to diversify host genetics [45, 46]. Our study emphasises the role of rotations in the long-term deployment of host resistance genes [47], improving management practices for the delay of pathogen evolution.

While our model currently analyses a monoculture in a single field per season, it could be extended by including more variation in host types, spatial structure and between-field pathogen migration, complementing previous work [48, 49] with the crop rotations perspective. Also, an increase in the number of host types and the number of pathogens could lead to a model exhibiting complex, and even chaotic, dynamics [50], which would be interesting to investigate. However, the most crucial step to take next would be the adaptation of the model to a particular case study.

As discussed during the analysis of optimal sequences under different conditions, not all pathogens respond the same to crop rotations, mainly depending on their life cycle. When focusing on microorganisms, soil-borne pathogens are commonly affected by the rotation practices, many of them being fungi. An excellent example of crop disease with these conditions is the disease take-all of cereals [51], in which the fungi *Gaeumannomyces graminis var. tritici* causes root rot of the host plant, usually wheat or barley—cash crops in the model. For take-all, the use of a non-cereal crop as break crop—which would be in our model the cover crop—is useful for disease control. This particular crop-pathogen system is suitable for the theory as developed herein. Other crop diseases such as white mould, by *Sclerotinia sclerotiorum* [52], would be a worthwhile investigation. The rotation scheme adapted to different crop types which all provide yield, as in the typical rotation of oilseed rape (host) and wheat (non-host) thereby will resulting in a modification of the theory to fit a specific model system.

Finally, we look at cash yield as the total economic benefit, without studying the costs of crop cultivation, and we do not emphasise how each sequence affects the farmers’ seasonal benefit. Hence our model only loosely connects with the economics of agrosystems. Farmers’ economic investment could be examined by including costs for each crop type. The short-term seasonal benefit can be regarded by simulating simultaneous rotation patterns for multiple subfields, with a minimum number of cash crops per season, which would assure the yearly economic return and alleviate concerns over the discount-rate [53]. Additionally, the rational application of non-host crops can reduce the use of pesticides, and the associated balance of economic costs is a whole socio-economic project in itself which we aim to connect with evolutionary biology in the future.

### Conclusion

Overall, our model can advise on strategies for maximising the gain of yield in cash crops, while using cover crops for soil improvement and control of pathogen spread. Further insights on rational resistance patterns could lead to new approaches for reducing pesticide use. Instead of applying the pesticide in all host seasons, the application could be limited to the host seasons where the pathogen density is low. This approach could both improve the efficiency of pesticides by increasing pest clearance and reducing the amount of pesticide used. Thus a synergistic use of crop rotations and pesticides can be possible together with biological control [54]. Experimental settings focus on crop rotations as the main factor for pest control, when put together with resistance variants and pesticides [25]. Agroecosystems rely on artificial selection for controlling the outcome of the harvest. We can profit from an evolutionary outlook bringing new tools towards more sustainable farming. Ideas such as the one presented in this research are essential first steps towards achieving this goal.

## Supporting information

S1 Appendix Description of best sequences under different soil contribution (*β*_*i*_), different crop types and justification of fixed parameters. (PDF)

S1 Fig Mode value of cash crops in the best rotation sequences for different values of soil contribution in cash and cover crops (*β*_1_,*β*_2_). The heat map shows the mode value of cash crops in the selection of ten best rotation sequences for maximum yield in absence of infection. Each patch represents a combination of a value of *β*_1_ ∈ {0, −0.5, −1, −1.5, −2} and a value of *β*_2_ ∈ {0, 0.5, 1, 1.5, 2}. The combination of values used for the results in the main text (*β*_1_ = −1.5, *β*_2_ = 1) is highlighted with a black square.
(TIF)
