# Supplementary material for: Eco-evolutionary agriculture: Host-pathogen dynamics in crop rotations
Source: PLoS Comput Biol. 2020 Jan 16;16(1):e1007546. doi: 10.1371/journal.pcbi.1007546 (PMC6964815; doi:10.1371/journal.pcbi.1007546)
Supplement: S1 Appendix — (PDF) [file pcbi.1007546.s001.pdf]

Supplementary Information: Eco-evolutionary agriculture: host-pathogen dynamics in crop rotations

Maria Bargués-Ribera[1], Chaitanya S. Gokhale[1*]

[1]Research Group for Theoretical Models of Eco-evolutionary Dynamics, Department of Evolutionary Theory, Max Planck Institute for Evolutionary Biology, August-Thienemann-Straße 2, 24306, Plön, Germany

*gokhale@evolbio.mpg.de

Best sequences under different soil contribution (β_i).

The parameter (β_i) captures how the planted crop affects soil quality. If cover crops improve soil quality rapidly and cash crops decrease it slowly, we would expect that optimal patterns have few cover crops, compared to the number of cash crops. In our case of study, we set $\beta_1 = -1.5$ for cash crops (c_1) and $\beta_2 = 1$ for cover crops (c_2), making soil quality decrease faster when one cash crop is cultivated, than the increase cover crops bring during one season.

Here, we analyse how the β parameter space changes the ratio of cash and cover crops in the selection of rotation sequences which perform best on maximising cash yield in the pathogen-free scenario. We explore the cash:cover ratio for the combinations of values $\beta_1 \in \{0, -0.5, -1, -1.5, -2\}, \beta_2 \in \{0, 0.5, 1, 1.5, 2\}$. The mode values of the selection for each combination of β values is shown in a heatmap (S1 Figure).

When $\beta_1 = 0$, for all β_2 values we get a high proportion of cash crops, compared to cover, in the selection of optimal sequences (number of cash crops ≥ 8). For $\beta_1 = -0.5$, the number of cash crops is equal or greater than the number of cover crops for all β_2 values (number of cash crops ≥ 5). If one season of cash or cover has the same (nonzero) magnitude of effect on the soil quality ($\beta_1 = \beta_2$) then we get always a 1:1 ratio of crops. Other combinations vary in the ratio, being the lowest a number of 3 cash crops when $\beta_1 = -1.5$ and $\beta_2 = 0.5$. For $\beta_2 = 0$ without infection, the best strategy is always to cultivate cash crops: even if we deplete the soil quality, the cash gain is greater than 0 (being the minimum soil quality $q(t) = 0.01$). The ten best sequences would then consist of one all cash sequence and nine sequences with one cover crop at different seasons. Thus overall, all combinations with $\beta_2 = 0$ show a mode value of 9 cash crops.

Characterisation of different types of crops.

In the main article, we have used two types of crop, designed as cover and cash crops and given specific parameter values. However, we can characterise a diversity of crops by using the parameters of soil contribution (β_i) and cash contribution (γ_i). Typically cover crops are defined as the crops that help increase the soil quality (positive β_i). These cover crops are often not directly related to the crop budget or do not have a direct payoff – in our model – low values of cash contribution (γ_i).

Cash crops are characterised by resulting high cash contribution (γ_i). However, cash crops usually deplete the soil of nutrients (negative values of soil contribution β_i) – creating a trade-off between the parameters. Picturing the crops in a space defined by the two parameters β_i and γ_i, those regions with high positive values for both would

appear empty due to these trade-offs, and those regions with negative values of β_i and null γ_i would not belong to crops of interest.

Finally, not only the soil contribution (β_i) and cash contribution (γ_i) characterise crops, but also the infectivity σ_i that the pathogen has for them. This dependence provides the opportunity to study further crop combinations with, for example, cash variants which have resistance to the pathogen – lowering σ_i – and pay a yield cost for such resistance – decrease in γ_i. Similarly, the pathogen could have different fitness values for both the cash crop and the cover crop – which could be replaced by a host crop with partial resistance ($w_{j2} > 0$).

Fixed parameters.

In the model, there are several parameters which have fixed values. We have compiled them in Table 1 (main text). Besides the infectivity and the death rate of the pathogen, which have the same values as in previous models, we have set the values for the rest. The soil (β_i) and cash (γ_i) contribution of the cash (c_1) and cover (c_2) crops have values according to the qualitative effect of their type of crop. During the same period, a cash crop decreases soil quality faster than a cover crop can recover it; and cover crops do not provide cash yield. The soil quality can be improved only up to a carrying capacity of K. The relative values of K and β_2 are chosen so as to reflect field observations (after four seasons of cover crops, the change in soil quality is not noticeable [1]). The pathogen strain transition rate μ has a high value due to the quick adaptation of pathogens to hosts in agro-ecosystems [2]. The initial host density has an arbitrary value, but this does not affect the optimality of the sequences because the crop loss is proportional to the initial host density.

References

1. de Boer HC, van Eekeren NJM, Pinxterhuis JB, Stienezen MWJ. Optimal length of the grass-clover period in crop rotations: results of a 9-year field experiment under organic conditions. Wageningen UR Livestock Research; 2012. 660.

2. McDonald BA, Stukenbrock EH. Rapid emergence of pathogens in agro-ecosystems: global threats to agricultural sustainability and food security. Philosophical Transactions of the Royal Society B: Biological Sciences. 2016;371(1709):20160026.
