# Supplementary figures and images for: Eco-evolutionary agriculture: Host-pathogen dynamics in crop rotations
Source: PLoS Comput Biol. 2020 Jan 16;16(1):e1007546. doi: 10.1371/journal.pcbi.1007546 (PMC6964815; doi:10.1371/journal.pcbi.1007546)

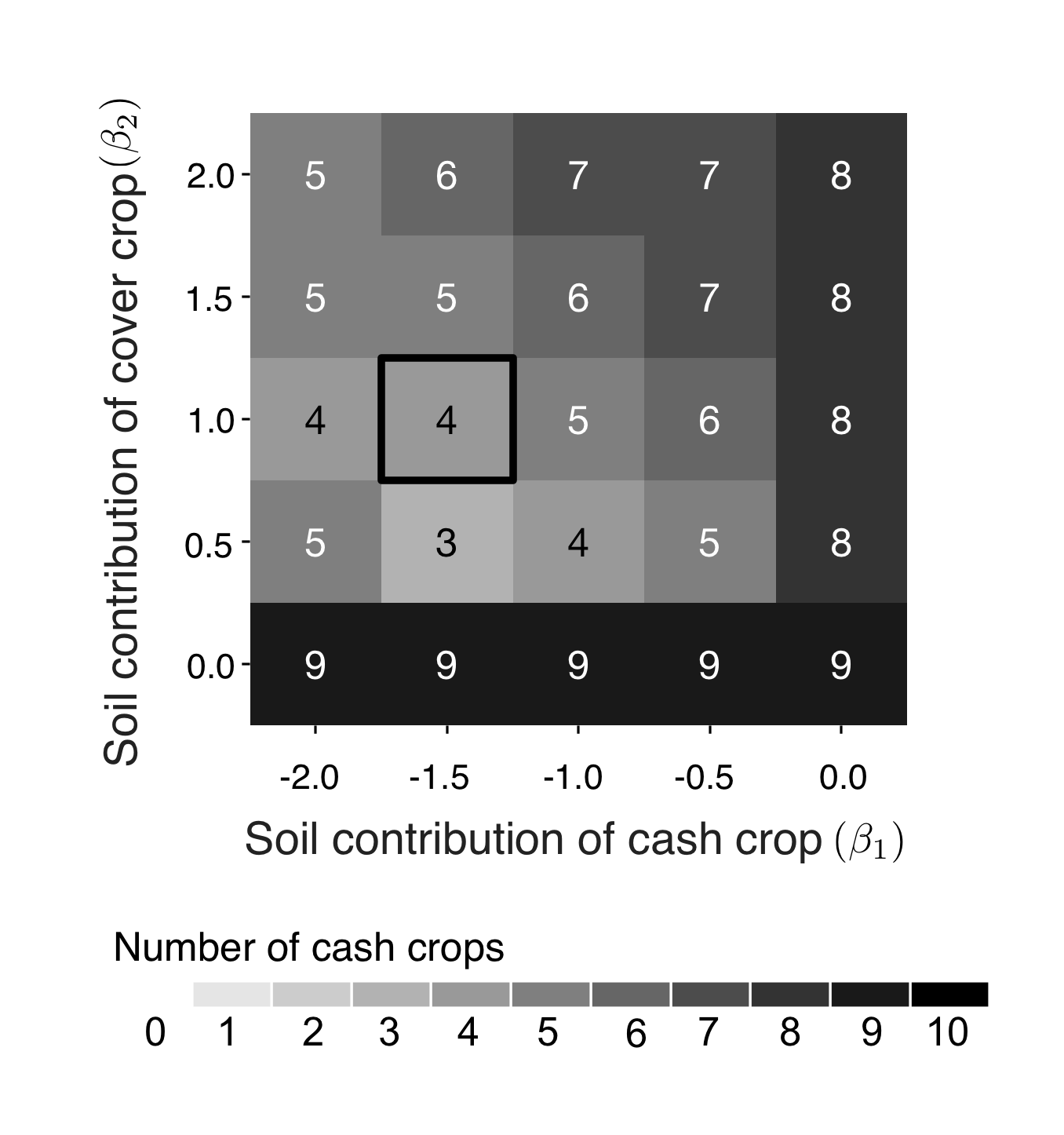

Supplement: S1 Fig — The heat map shows the mode value of cash crops in the selection of ten best rotation sequences for maximum yield in absence of infection. Each patch represents a combination of a value of β1 ∈ {0, −0.5, −1, −1.5, −2} and a value of β2 ∈ {0, 0.5, 1, 1.5, 2}. The combination of values used for the results in the main text (β1 = −1.5, β2 = 1) is highlighted with a black square. (TIF) [file pcbi.1007546.s002.tif]
